# The Incentive-Sensitization Theory of Addiction 30 Years On

**DOI:** 10.1146/annurev-psych-011624-024031

**Published:** 2024-12-03

**Authors:** Terry E. Robinson, Kent C. Berridge

**Affiliations:** Department of Psychology, University of Michigan, Ann Arbor, Michigan, USA

**Keywords:** addiction, sensitization, dopamine, motivation, wanting, craving, liking

## Abstract

The incentive-sensitization theory (IST) of addiction was first published in 1993, proposing that (*a*) brain mesolimbic dopamine systems mediate incentive motivation (“wanting”) for addictive drugs and other rewards, but not their hedonic impact (liking) when consumed; and (*b*) some individuals are vulnerable to drug-induced long-lasting sensitization of mesolimbic systems, which selectively amplifies their “wanting” for drugs without increasing their liking of the same drugs. Here we describe the origins of IST and evaluate its status 30 years on. We compare IST to other theories of addiction, including opponent-process theories, habit theories of addiction, and prefrontal cortical dysfunction theories of impaired impulse control. We also address critiques of IST that have been raised over the years, such as whether craving is important in addiction and whether addiction can ever be characterized as compulsive. Finally, we discuss several contemporary phenomena, including the potential role of incentive sensitization in behavioral addictions, the emergence of addiction-like dopamine dysregulation syndrome in medicated Parkinson’s patients, the role of attentional capture and approach tendencies, and the role of uncertainty in incentive motivation.

## 1. INTRODUCTION

For many years the most widely accepted explanations of drug addiction were that people initially take drugs because their effects are pleasant, and later, with the development of tolerance, keep taking drugs to avoid negative distress feelings caused by withdrawal. However, it was also known that there were problems with these explanations for addiction. For example, people often take drugs that are not strongly pleasurable (e.g., nicotine) and can become addicted to drugs that cause only weak withdrawal (e.g., cocaine). Further, it is common for people to complete detoxification programs, and so escape from withdrawal distress, and to yet relapse back into drug taking when they return to the outside world, sometimes months or years after they stopped using drugs. Thus, traditional explanations of addiction leave several important phenomena unexplained.

We proposed the incentive-sensitization theory (IST) of addiction in 1993 in part to fill that explanatory gap, and in part based on what were then new laboratory discoveries (Robinson & Berridge 1993). In the ensuing 30 years, our original paper has been cited over 9,150 times (based on Google Scholar) suggesting it has attracted considerable interest and debate. Here we describe the theory’s origins, consider alternative views of addiction, address critiques that have been raised over the years, and discuss some current issues.

## 2. THE ORIGIN OF THE INCENTIVE-SENSITIZATION THEORY

### 2.1. The Discovery That Drugs Cause Mesolimbic Sensitization

Prior to the 1980s, most brain-related theories of addiction focused on the development of drug-induced tolerance with repeated use, a decrease in a drug effect over time, and the physiological and psychological expression of withdrawal when drug use was stopped. This was in part because many studies at the time focused on opioids and alcohol, which produce strong tolerance and withdrawal, and it was thought that the distress associated with withdrawal provided the primary motivational force in addiction (Solomon & Corbit 1974). However, in the 1980s more studies started to appear on a surprisingly different phenomenon: drug-induced sensitization, which refers to an increase in a drug effect with repeated drug use (for reviews, see Post 1980, Robinson & Becker 1986, Kalivas & Stewart 1991). Although several drug effects may sensitize, most early studies focused on psychomotor effects (e.g., hyperlocomotion, stereotyped behaviors) produced by amphetamine, cocaine, and other psychostimulant drugs, although the psychomotor effects of opioids, alcohol, and other potentially addictive drugs were also shown to sensitize. Psychomotor effects were studied because they were thought to reflect mesostriatal dopamine system activation, and because they were relatively easy to quantify in animals. These studies soon revealed large individual differences in susceptibility to drug sensitization—influenced by an individual’s genes, sex, hormonal state, previous stress/trauma experiences, the environmental context in which drugs were taken, etc.—and the importance of intermittent administration (Post 1980, Robinson & Becker 1986, Segal & Kuczenski 1987). Critically, it was found that once sensitization was induced in a vulnerable individual, it was extremely long-lasting, persisting for months to years in both rats (Bartoletti et al. 1983, Paulson et al. 1991) and people, even after drug use was stopped (Boileau et al. 2006).

At the time it had been established that the psychomotor effects of psychostimulant drugs were mediated by their activation of mesostriatal dopamine systems. Accordingly, it was shown that drug-induced psychomotor sensitization was associated with increases in the amount of mesostriatal dopamine release induced by drugs in isolated brain slices (Robinson & Becker 1982, Kolta et al. 1985) and intact animals (Ichikawa 1988, Robinson et al. 1988).

### 2.2. The Role of Dopamine in Reward: “Wanting” or Liking?

By the 1980s it was known that mesolimbic dopamine projections were also a chief brain mechanism of reward, including drug reward (Wise 1985, Fibiger & Phillips 1986, Wise & Bozarth 1987). The dominant theory at the time was that the role of dopamine in reward was to mediate the hedonic pleasure, or liking, produced by drugs, foods, sex, etc. (Wise 1985). Researchers also proposed that learned drug cues could evoke dopamine release that produced pleasure as a conditioned response and consequently increase incentive motivation to seek drugs and cause relapse (Stewart et al. 1984). The prevalence of the dopamine = pleasure hypothesis was probably the reason that early theorists (including ourselves) did not think at first that drug-induced mesolimbic sensitization was relevant to addiction. After all, if dopamine caused pleasure, then dopamine sensitization should make addicts like their drugs more—so that they might be able to take less drug yet still get the same enjoyment. No one thought then or now that “more pleasure, less drug” was an accurate description of addiction (for review, see Phillips & Ahn 2022).

Initially, we too believed the dopamine = pleasure hypothesis. However, our thinking began to change based on the results of several experiments in animals led by one of us on dopamine’s role in taste pleasure (Berridge et al. 1989, Treit & Berridge 1990, Berridge & Valenstein 1991). Those studies relied on an unconditioned measure of the hedonic (“liking”) effects of food tastes: a taste reactivity measure of immediate hedonic impact reflected in affective facial expressions, similar to how human parents might determine whether their infant enjoys the taste of the family’s food. Positive “liking” expressions (e.g., licking of the lips) are elicited from infants by sweet tastes, whereas negative disgust expressions (e.g., gapes) are elicited by bitter tastes (Steiner 1973). Apes, monkeys, and even rats show similar taste-elicited facial expressions of sweet “liking” versus bitter disgust (Grill & Norgren 1978, Berridge 2000, Steiner et al. 2001).

If dopamine mediated the pleasure of taste liking, we expected drugs that block dopamine receptors to suppress rats’ facial “liking” reactions to sweet tastes and possibly to increase disgust expressions. To our surprise, dopamine antagonists did not change “liking” reactions to sweetness (Treit & Berridge 1990, Peciña et al. 1997), nor did the near-total destruction of mesolimbic and mesostriatal dopamine projections produced by brain microinjections of the neurotoxin 6-hydroxydopamine (Berridge et al. 1989, Berridge & Robinson 1998). For example, rats in which 98–99% of nucleus accumbens and dorsal striatum dopamine was depleted never voluntarily ate or drank or pursued any other reward, but they still had normal “liking” reactions to a sweet solution infused into their mouth. Following taste aversion training dopamine-depleted rats were also able to learn a new disliked value of the formerly “liked” sweet taste, now emitting disgust gapes, headshakes, etc. Finally, it was shown that the aphagic rats would increase their “liking” reactions to a different sweet taste if given a nondopaminergic drug that enhanced palatability. That pattern led us to suggest for the first time that mesostriatal dopamine was not needed to mediate “liking” for sweet rewards after all, but dopamine was instead necessary to “want” rewards that remained “liked” (Berridge et al. 1989). Indeed, there were already other clues in the literature at that time that dopamine might have a role more relevant to “wanting” in addiction (for review, see Phillips & Ahn 2022).

Incentive salience was the name we gave to the form of motivational “wanting” mediated by dopamine, proposing in 1989, that “meso-striatal dopamine neurons belong to a system that assigns salience or motivational significance to the perception of intrinsically neutral events” (i.e., “wanting”), but that dopamine was not required “for biologically significant stimuli to engender affect” (i.e., “liking” or liking) (Berridge et al. 1989, pp. 42–43). Initially, the dopamine = “wanting” hypothesis was based only on the effects of dopamine suppression (see also Fibiger & Phillips 1986, Blackburn et al. 1987). Soon, however, we also found positive evidence that stimulation of mesolimbic dopamine systems in rats increased their motivational “wanting” to pursue or consume food rewards yet failed to enhance their “liking” of those same rewards (Berridge & Valenstein 1991, Wyvell & Berridge 2000). In 1989 we did not directly apply these findings to addiction, but they did start conversations between us that eventually led to the IST.

### 2.3. The Incentive-Sensitization Theory of Addiction

The logic of the IST of addiction is simple (Robinson & Berridge 1993). It just combines the three major findings described above: (*a*) Repeated intermittent exposure to addictive drugs produces mesolimbic sensitization in vulnerable individuals, causing an increase in drug-induced dopamine release, and related neural changes; (*b*) once induced, mesolimbic sensitization is very persistent, lasting long after drug use is discontinued; and (*c*) the role of dopamine in reward is to mediate incentive salience or “wanting”, especially when triggered by reward cues, but not to mediate hedonic pleasure or liking of rewards. Sensitized “wanting” can become narrowly focused on drugs in particular, via associative pairings of drug cues and drug taking with mesolimbic activations. From these three points it follows logically that with repeated exposure a susceptible individual would experience a progressive and persistent increase in cue-triggered “wanting” for drugs, regardless of whether drug liking changes or even declines. This explains why excessive motivation for drugs grows disproportionately to the pleasure drugs produce as addiction develops, and the persistence of sensitized “wanting” explains why susceptibility to relapse can persist long after the discontinuation of drug use.

Importantly, a sensitized dopamine system is not always hyperactive, but it is phasically hyperreactive to drug cues and contexts that can trigger “wanting” to take drugs. This leads to phasic spurts of intensified desire at particular moments, especially when drug cues are encountered. Beyond physical drug cues, vivid drug-related imagery can also trigger “wanting” in people. Additionally, the contexts in which drugs have been experienced powerfully gate the ability of drugs or cues to elicit sensitized reactions (Anagnostaras et al. 2002). Finally, sensitized “wanting” can become even stronger in transient states of stress, appetites, emotional excitement, withdrawal, and so on, that increase mesolimbic reactivity to a reward cue (Wemm et al. 2022, Berridge 2023). This was the essence of IST in 1993 and remains so today.

Of course, there are many other reasons people initially take drugs or even go on to abuse them: to fit in socially, to enjoy drug pleasure, to reduce distress or anxiety, and so on. For many people, these reasons may fully account for their drug use, and most may never become sensitized or addicted in a compulsive sense. They may remain able to quit drugs if they choose. For example, among those who try heroin, only 25–40% become addicted (Santiago Rivera et al. 2018), and under 4% of those medically prescribed opioids for pain relief go on to develop problematic drug use (Epstein et al. 2018). Of those who do abuse drugs in their late teens or 20s, many stop on their own by their 30s or 40s. Many such people may have never developed robust incentive sensitization.

Incentive sensitization is specifically meant to explain the essence of arguably compulsive addiction in those who are vulnerable, namely, intense urges to take drugs even in the face of dire negative consequences and often despite a sincere desire to quit. These urges to take drugs can persist even if the person has stopped taking drugs for months or years, in the absence of distress or withdrawal, and even if the person does not expect to like the drugs much anymore. Indeed, several recent studies in people have confirmed that in addiction “wanting” can dissociate from liking (Koranyi et al. 2017, Grigutsch et al. 2019, King et al. 2021). In short, IST helps explain why in vulnerable individuals drug use can transition to levels that seem irrational and compulsive (see below).

It seems fair to say that IST was not readily embraced at first, probably for several reasons. For example, the earliest studies on behavioral sensitization measured psychomotor activation in animals, which could be easily discounted as not being relevant to addiction, despite the fact that later animal studies showed incentive sensitization also increased behavioral “wanting” for rewards, and some authors argued that the neural bases of psychomotor activation and reward motivation partly overlap (e.g., Wise & Bozarth 1987). Also, the idea that dopamine mediated hedonic liking was a powerful meme, both in the popular imagination and among neuroscientists, and continued to dominate discourse for years, even up to the present day (see below).

However, in the 2000s two other developments contributed to a shift in thinking about the role of dopamine in reward. One was the discovery that dopamine neurons are often more highly activated by learned cues that predict rewards than by the rewards themselves (Schultz et al. 1997). The second was the emergence of studies in humans showing that dopamine suppression reduces subjective wanting but not liking of rewards, including drug rewards (Brauer & De Wit 1997; Leyton et al. 2005, 2007; Leyton 2010). Furthermore, so-called anhedonic Parkinson’s patients, depleted of dopamine, were found to still give perfectly normal ratings of liking for sugar and chocolate or vanilla milkshakes (Sienkiewicz-Jarosz et al. 2005, 2013), even when the patients were apathetic toward those and other rewards. Gradually, the field moved toward our conclusion that dopamine mediates “wanting” for rewards but not liking for the same rewards (Salamone & Correa 2012).

Yet it would be rash to conclude that the dopamine = pleasure meme has faded entirely from the field. Occasionally, even prominent addiction neuroscientists still lapse back into phrases that can only be understood as supposing dopamine to be a primary mechanism of drug pleasure. For example, Volkow and colleagues wrote in 2016 that “this attenuated release of dopamine renders the brain’s reward system much less sensitive to stimulation by both drug-related and nondrug-related rewards. As a result, persons with addiction no longer experience the same degree of euphoria from a drug as they did when they first started using it,” and “the down-regulation of dopamine signaling…dulls the reward circuits’ sensitivity to pleasure” (Volkow et al. 2016, p. 366; but see Compton et al. 2022). Similarly, Koob (2021, p. 173) recently wrote that “a prominent neuroadaptation is the loss of dopaminergic function in the mesolimbic dopamine pathway, which is hypothesized to contribute to hedonic tolerance,” that is, to diminish drug pleasure. Such postulated reductions in capacity for pleasure/positive reinforcement when dopamine is suppressed have also been called a reward or dopamine deficiency syndrome (Blum et al. 1996, Kotyuk et al. 2022). Yet, by and large, affective neuroscientists have mostly moved on from the view that dopamine causes pleasure.

## 3. ROLES OF CRAVING IN ADDICTION AND INCENTIVE SENSITIZATION

The title of our original 1993 article began with “The Neural Basis of Drug Craving,” so some comment on the role of craving in addiction is warranted, as it has been controversial (e.g., Tiffany 1990). On one hand, drug taking and relapse can sometimes occur in the absence of strong subjective craving (Sripada 2022). On the other hand, strong feelings of craving often do accompany relapse (Preston et al. 2009, Marhe et al. 2013, Sayette 2016, Koban et al. 2022, Vafaie & Kober 2022). For example, a recent meta-analysis of craving reported that “a 1-unit increase in cue and craving indicators was associated with more than double the odds of future drug use and relapse,” and its authors concluded that “cues and craving may reliably predict drug use and relapse outcomes and may be core mechanisms underlying drug use” (Vafaie & Kober 2022, p. 641). Subjective craving is also associated with greater capture of visual attention by alcohol cues in people with alcohol use disorder, consistent with higher incentive salience (Bollen et al. 2024).

Incentive salience can often give strong urgency to conscious feelings of craving (Robinson & Berridge 1993), but as we cautioned in 1993, incentive salience can also occur unconsciously in some situations and yet still motivate behavior (Lamb et al. 1991, Fischman & Foltin 1992, Winkielman et al. 2005, Wiers et al. 2021). Unconscious incentive salience can even control desire in ordinary human adults. For example, presenting subliminally brief visual flashes of happy or angry facial expressions can modulate the intensity of wanting for a subsequent beverage, even if the person fails to consciously perceive the faces and fails to detect their own affective reactions (Winkielman et al. 2005). Similarly, subliminal drug or other reward cues can activate brain mesocorticolimbic circuits in the absence of conscious awareness of the cues (Childress et al. 2008, Wiers et al. 2021). Unconscious incentive salience may explain why drug taking and relapse sometimes can occur in the absence of any strong subjective craving.

A vivid description of unconscious “wanting” for drugs in addiction was provided by Fischman & Foltin (1992), who allowed cocaine users to earn intravenous cocaine infusions by pressing either a button that delivered a dose of cocaine or another button that delivered either a different dose of cocaine or saline. On a particular day, only saline versus a very low dose of cocaine was available. The cocaine dose was so low that it failed to produce any subjective feelings of pleasure or arousal or any detectable cardiovascular effects, and the cocaine users reported that on that day both buttons were worthless and both delivered only saline (Fischman & Foltin 1992). Yet they still repeatedly pressed the low-dose cocaine button more than saline. When Fischman was asked to reconcile how users could report no cocaine effects yet still work specifically for the drug, she replied, “If you want to know what the subjects say about their self-administration of these low doses, they tell me that they were not choosing cocaine over placebo. They often insist that they were sampling equally from each of the two choice options and both were placebo. On the other hand, if you look at the data from that session, you see that they were choosing (cocaine)” (see Fischman & Foltin 1992, p. 179). A similar study reported that opioid users worked for a very low dose of morphine, despite saying that it had no subjective effects and was placebo (although they did not work for actual placebo) (Lamb et al. 1991). These authors concluded that “the reinforcing effects of morphine can occur in the absence of self-reported subjective effects and thus, do not appear to be causally related to drug-liking or euphoria” (Lamb et al. 1991, p. 1172). In our view, such examples demonstrate objective “wanting” in the absence of conscious desire for the chosen target as well as in the absence of conscious pleasure.

In summary, subjective craving plays a prominent role in addiction, but it is not always necessary to motivate drug-seeking. We suspect that the role of implicit incentive salience in addiction deserves more attention (Wiers et al. 2021).

## 4. RELATION TO OTHER THEORIES OF ADDICTION

### 4.1. The Role of Withdrawal Distress: Opponent-Process Theories

As mentioned above, a dominant view of addiction in the twentieth century was that people start using drugs for pleasure (or self-medication of preexisting distress), but addiction develops only after drug taking comes to be motivated by the desire to avoid aversive withdrawal feelings. This withdrawal-centric view was formalized by opponent process theory, which posited that addictive drugs initially generate a pleasant a-process, followed by an opposing negatively valenced b-process as a form of negative feedback to restore hedonic balance (Solomon & Corbit 1974). The b-process was hypothesized to be initially weaker than the a-process, but with repeated drug use the b-process was thought to selectively grow progressively in amplitude and duration, while the a-process remained unchanged. The strengthened b-process eventually resulted in tolerance and weaker pleasure while drug was on board and unpleasant withdrawal feelings after drug ended.

Beginning in the 1990s, Koob and colleagues suggested brain mechanisms that could mediate opponent a- and b-processes (Koob & Le Moal 1997, 2001; Volkow et al. 2016; Koob 2021). The pleasant a-process was initially posited to be generated by the drug’s activation of mesolimbic dopamine and opioid neurons, and the unpleasant b-process by activation of stress-related corticotropin releasing factor (CRF) and related neurotransmitter systems in the extended amygdala. This theory has had several names over the years (hedonic homeostasis, hedonic dysregulation, allostasis, and hyperkatifeia), but its basic tenets have remained essentially the same (Koob 2021). When reduced to their core, all these versions posit that the primary motivational force to take drugs in addiction is avoidance of unpleasant withdrawal and other distress feelings, maintained by negative reinforcement (Koob 2021).

We agree that many people with addiction take drugs to avoid unpleasant feelings of withdrawal or other distress. We question, however, whether withdrawal/distress is the primary problem in addiction, joining many others who have similarly questioned the necessity of distress in addiction (e.g., Wise & Bozarth 1987, Robinson & Berridge 1993, Wise & Koob 2014, Berridge & Robinson 2016, Martinez et al. 2022). If addiction were due primarily to avoiding distress, then relieving distress would eliminate addiction. However, addiction can occur even in users who do not experience strong or lasting withdrawal symptoms when stopping their drugs, and many cases of relapse occur in the absence of distress. For example, a study of people who used cocaine reported that “the symptomatology resulting from abstinence following pharmacological exposure to high doses of cocaine may be of modest severity and fairly short-lived,” and there is “no evidence of protracted mood or biological disruptions during the longer 28-day observation period” (Walsh et al. 2009, p. 205). Similarly, although alcohol can induce strong withdrawal in some heavy users, other “AUD [alcohol use disorder] drinkers continue to experience pleasurable effects of alcohol, which may contribute to the excessive drinking that is characteristic of human AUD at a stage without significant negative affect or withdrawal symptoms” (King et al. 2022, p. 1898). Of course, several physiological states can amplify the intensity of incentive salience attribution (Berridge 2023), and drug withdrawal may be one such state: For example, opioid withdrawal is reported to both increase the incentive value of drug cues and amplify cue-triggered brain activations (Shi et al. 2021). So, beyond distress, withdrawal may also contribute to drug taking by increasing incentive motivation or “wanting” for drugs (Wise & Bozarth 1987, Robinson & Berridge 1993, Wise & Koob 2014, Berridge & Robinson 2016). Thus, despite being a factor in motivating drug taking, withdrawal feelings are neither necessary nor sufficient for inducing compulsive addiction or relapse.

#### 4.1.1. Roles of corticotropin releasing factor in addiction.

Any discussion of opponent processes and distress should also consider the roles of CRF neurotransmission in addiction. Koob and colleagues (Koob et al. 2020, Koob 2021) proposed that the chief b-process mechanism for distress feelings is mediated by the release of CRF in the extended amygdala (i.e., central nucleus of the amygdala plus bed nucleus of the stria terminalis) (Koob & Le Moal 1997, 2001; Volkow et al. 2016; Koob et al. 2020; Koob 2021). CRF release is indeed triggered by unpleasant stressors, but CRF release in central amygdala is also triggered by pleasant rewards, such as food, in the absence of any distress (Merali et al. 1998). Further, stimulating CRF signals in amygdala or nucleus accumbens can increase incentive motivation to pursue food or drug rewards without inducing distress, similar to the effect of activating dopamine (Peciña et al. 2006, Lemos et al. 2012, Baumgartner et al. 2022). Lack of distress is shown by observations that the same rats do not try to avoid CRF stimulation, but if anything, they seek it out (Peciña et al. 2006, Lemos et al. 2012, Baumgartner et al. 2022). Such findings have raised the alternative possibility that CRF in limbic structures can sometimes promote incentive salience in the absence of distress. Thus, if CRF release contributes to relapse, it may do so either by amplifying “wanting” or as a consequence of distress, depending on other factors. This may be why even happy stresses such as a promotion or winning the lottery are said to increase relapse in some addicted individuals (Annis & Graham 1995, Larimer et al. 1999) without involving an aversive b-process.

#### 4.1.2. Dopamine: up or down in addiction?

Addiction has been posited by some to be due primarily to a hypodopaminergic state (e.g., loss of pleasure or liking, reward deficiency) and by us and others to be due instead to a hyperreactive dopamine state (excessive “wanting” elicited by drug cues). This complicated issue has been discussed previously (Leyton & Vezina 2012, 2014; Leyton 2021; Samaha et al. 2021), so we make only a few points here. First, evidence for a hypodopaminergic state reported in studies on addicted humans is subject to alternative interpretations (e.g., see Samaha et al. 2021, box 2; see also Jangard et al. 2023). For example, a decrease in dopamine D2 receptors [inferred from reduced D2 binding in positron emission topography (PET) studies] is often cited as evidence for a dopamine deficiency (Volkow et al. 2016, Ashok et al. 2017, Koob 2021). However, rather than causing a person to become addicted, D2 receptor downregulation may instead occur later in addiction as a consequence of heavy drug use. That is, high levels of dopamine release, caused by taking dopamine-promoting drugs, can cause neurons to downregulate their dopamine D2 receptors as a partial compensatory response (Darcey et al. 2023). Compensatory downregulation would be a consequence, not a cause, of the addiction.

Second, some studies have failed to find evidence for downregulation of D2 receptors in people with addiction (for reviews, see Leyton 2021, Samaha et al. 2021). This raises doubts about the universality of downregulation. Further, even studies that do find D2 downregulation often still report that the same addicted individuals show drug cue–triggered limbic hyperactivations that are consistent with incentive sensitization, not deficiency (Moeller et al. 2013, Koob & Volkow 2016, Volkow et al. 2016, Zilverstand & Goldstein 2020, Huang et al. 2024). Drug cues or vivid imagery of drug taking are chief triggers of incentive salience, so cue-triggered mesolimbic hyperactivation suggests excessive wanting in people with addiction.

Third, the presence versus absence of drug-associated contexts in functional magnetic resonance imaging (fMRI) and PET studies may partly explain the fact that some studies report sensitized hyperreactivity of dopamine release in addiction, whereas others report mesolimbic suppression. Drug contexts gate the ability of drug cues to trigger the expression of sensitization: Sensitized mesolimbic reactions are often seen only when a drug or its cue is presented in a drug-associated context, not when the same cue is presented in a non-drug context (Robinson et al. 1998, Guillory et al. 2022). This appears to be due, in part, to an inhibitory occasion-setting process in non-drug contexts (Anagnostaras et al. 2002, Guillory et al. 2022), by which a stimulus or context that is not associated with reward can inhibit the expression of sensitized behavioral and dopamine responses to a drug reward. As put by Guillory et al. (2022, p. 8), “Conditioned inhibition could explain why detoxified volunteers with a cocaine use disorder have reduced striatal DA [dopamine] responses following a psychostimulant challenge.… [t]he laboratory setting is not associated with drugs and thus functions as a conditioned inhibitor (Vezina & Leyton 2009).” For example, flight attendants who are smokers typically do not experience cigarette craving on a long flight, presumably because the airplane predicts drug unavailability; craving only arises toward the end of the flight, which signals it will soon be possible to smoke (Dar et al. 2010). In support of this view, sensitized mesolimbic dopamine responses to cocaine are facilitated when human cocaine users are allowed to prepare cocaine with their usual paraphernalia and procedures (Cox et al. 2009) but not when those drug paraphernalia are missing (Casey et al. 2014). Overall, the more life-like the experience of drug taking in a neuroimaging scanner, the more likely the detection of cue-triggered hyperreactivity in mesolimbic wanting systems (Vezina & Leyton 2009, Leyton & Vezina 2012, Samaha et al. 2021). This may also be why “Cocaine craving is tightly coupled to cocaine use in users’ normal environments” (Preston et al. 2009, p. 291), especially under conditions of stress (Preston et al. 2018), and is weaker when in an unfamiliar context or intimidating laboratory (Preston et al. 2009).

### 4.2. Habit Theories of Addiction

Used purely as a descriptive label, the notion of habit surely fits addiction: After all, drugs are taken again and again. However, to make it into an explanation for addiction, habit must be defined in a way that gives it explanatory force. Perhaps the clearest explanatory definition of habit is also the oldest. Namely, habits were posited to be automatic stimulus-response (S-R) chains that surface especially when a person’s attention was distracted (James 1890). For example, Guthrie (1935, p. 139) used this notion of habit to explain smoking relapse: “I had once a caller to whom I was explaining that the apple I had just finished was a splendid device for avoiding a smoke. The caller pointed out that I was at that moment smoking. The habit of lighting a cigarette was so attached to the finish of eating that smoking had started automatically.” In other words, addictive habits took over in moments of inattention. Similarly, repeated drug taking has been proposed to lead to the development of automatized action schemata (Tiffany 1990).

Some modern habit theories of addiction use a different definition that is less explanatory because it is merely a negative definition that specifies what habits lack rather than what they are. These modern habit theories typically dichotomize all reward seeking as being either goal-directed actions (i.e., sensitive to the current value of the goal) or actions that are not goal-directed (i.e., persisting when the goal is devalued), and they assign the habit label to all seeking behavior that persists after a goal is devalued (Everitt et al. 2008, 2018; Everitt & Robbins 2016; Wood & Runger 2016; Lüscher et al. 2020). The problem with this negative definition, however, is that there are many alternative psychological reasons that could also cause a response to persist after goal devaluation, including a motivational compulsion due to sensitized incentive salience (Robinson & Berridge 1993, Berridge & Robinson 2016). Even Tolman (1955), who originated this approach to identify goal-directed actions, did not label actions that persisted after goal devaluation as habits but rather suggested that persistence reflected a distortion of cognitive expectations due to overtraining, which in Tolman’s words “narrow[s] the rat’s ‘cognitive maps’” (p. 36). In short, the dichotomy between goal-directed cognitive plans and nongoal-directed habits is artificial and too simplistic, leaving out other psychological processes that can cause pursuit to persist.

Perhaps recognizing this, some modern habit theorists have recently suggested additional positive features for habits. One suggestion is neuroanatomical: Namely, if the dorsolateral region of neostriatum (DLS) comes to control actions, then those actions are called habits, and this is proposed to happen as addiction develops (Everitt et al. 2008, Giuliano et al. 2019, Lüscher et al. 2020). The DLS is indeed involved in habitual sequences of learned movements as well as in other instinctive sequences of movements, sensory guidance of actions, direct initiation of some movements, and so on (Smith & Graybiel 2016, de la Torre-Martinez et al. 2023). However, the DLS also contributes to incentive motivation for rewards. For example, rodents will work for optogenetic stimulation of DLS neurons (Vicente et al. 2016). Further, neurochemical opioid or dopamine microinjections into the DLS increase the ability of reward cues to trigger “wanting” (DiFeliceantonio & Berridge 2016), and lesions of the DLS reduce cue-triggered wanting for rewards (Corbit & Janak 2007). In people with addiction, cocaine cues elicit high DLS activation and dopamine release, as do food cues in people who are obese and binge eaters (Volkow et al. 2006, Wang et al. 2011). Indeed, higher cue-triggered dopamine release in DLS corresponded to higher craving in people with cocaine addiction, leading Volkow and colleagues to propose that “it would appear as if DA activation of dorsal striatum is involved with the ‘desire’ (wanting), which would result in the readiness to engage in the behaviors necessary to procure the desired object” (Volkow et al. 2006, p. 6586). Higher dorsal striatal activation by alcohol cues is also associated with higher risk of relapse in alcohol use disorder (Bach et al. 2015). All this seems consistent with the hypothesis that DLS recruitment in addiction may further intensify the incentive motivational impact of reward cues, beyond any role the DLS may play in habits. Thus, DLS recruitment is ambiguous for defining a habit, as it could reflect equally well greater incentive motivation.

At a more psychological level, a habit theory should explain why addiction can seem compulsive. Yet the automatic S-R habit theory described by Guthrie to explain smoking relapse did not involve compulsion: Relapse happened only when attention was distracted. To add a compulsive feature to habits, Everitt and colleagues suggested that addictive habits take on a special “Must Do!” quality not possessed by ordinary habits (Everitt et al. 2008) and proposed the concept of incentive habits (Belin et al. 2009), which incorporates motivational processes. Indeed, others subsequently suggested the “Must Do!” feature would need to be motivational, so that the individual with addiction intensely wants to take the drug (Bechara et al. 2019, Berridge 2021, Vandaele & Ahmed 2021). By that interpretation, the explanatory burden for addictive compulsion shifts from habit per se to excessive motivation, similar to IST.

Habit theory advocates have given mixed replies to this suggestion that the “Must Do!” feature of an addictive habit involves intensified motivation. For example, Robbins dissented and suggested instead that “‘Must Do!’ responses may also be considered as possible rationalizations rather than motivational influences per se. Patients with Gilles de Tourette’s syndrome similarly have compulsive urges to perform certain behaviors, as a consequence of dysregulated corticostriatal circuitry; but these are probably not ‘motivated’” (Robbins 2019, p. 93). Robbins’s suggestion implies that addictive habits erupt involuntarily like a compulsive Tourette’s tic, without motivation to do so. But is drug seeking or relapse ever experienced as an involuntary motor tic? We have doubts. An alternative motivational interpretation of “Must Do!” habits has been offered recently by Fouyssac et al. (2022). Like Robbins (2019), they suggest that addictive habits are akin to Tourette’s movements, writing that “this urge to act…may resemble the motor urges characteristic of impulsive/compulsive disorders such as Tourette syndrome” (Fouyssac et al. 2022, p. 1055). However, they suggest this involves an aversive motivational component, namely, a distressing “negative state associated with the urge” if the movement is not performed (Fouyssac et al. 2022, p. 1056). They suggest that individuals with addiction perform the action of taking drugs to relieve distress produced by not performing the action. Seeking relief from distress is in a sense analogous to the aversive escape logic of opponent process theories (Solomon & Corbit 1974, Koob 2021). However, the chief difference is that Fouyssac et al. (2022) ascribe negative distress to the absence of the habitual drug-taking action rather than to the absence of the drug itself.

We agree with Everitt, Robbins, Fouyssac, and colleagues that an urge to perform drug-related actions, in addition to the urge for drug effects, may contribute to addictive drug taking. Indeed, an early neuroscience hypothesis of reinforcement suggested that performing instinctive consummatory actions was itself intrinsically motivating and rewarding (Glickman & Schiff 1967). However, the motivation to act need not be caused by distress. The words “movement” and “motivation” share both etymological origins and brain mechanisms, and it is probably no accident that dopamine is important for both: One function contributed by dopamine in nucleus accumbens and striatum may be the motivation to act (Salamone & Correa 2012). This is why 30 years ago we suggested the possibility that dopamine might amplify action salience, noting that brain dopamine systems might attribute incentive salience to brain representations of some actions as well as of stimuli. We wrote that “a psychological function of this (mesolimbic) neural system is to attribute incentive salience to the perception and mental representation of stimuli *and actions*” (Robinson & Berridge 1993, p. 266, emphasis added). That is also consistent with emotion theories positing that emotions contain an “action tendency” as an essential component (Frijda 1987), as well as with suggestions that dopamine contributes to motivation to play (Siviy & Panksepp 2011, Vanderschuren et al. 2016). Thus, we believe that people with addiction may sometimes “want” to perform drug-taking actions, whether the action is habitual or not, via incentive salience, much as they “want” drugs and drug cues.

Whatever the role of striatal dopamine in motivated action, we close with two final points about habits. First, in animal studies the development of habitual drug-seeking behavior (i.e., behavior that persists after goal devaluation) typically requires the use of specific test procedures, such as second-order schedules of reinforcement, which may not reflect the human drug user’s situation. Second, addiction is clearly not reducible to habits alone, as people with addiction often must devise new and flexible behavioral strategies to obtain what they seek. As Singer et al. (2018, p. 61) pointed out, “to procure drugs, addicts typically show considerable ingenuity and flexibility in their behavior, first, to acquire the money to purchase drugs, then to locate a possible drug source, and finally to negotiate a purchase, often under very challenging circumstances. … Such motivated, goal-directed behavior requires daily solutions to unique problems and, by definition, is not habitual.” Addicted individuals who innovate strategies to procure drugs are highly motivated, and we believe it is sensitized “wanting”, not a ritualized habit sequence, that poses the fundamental problem in addiction (also see Hogarth 2020).

### 4.3. Cortical Dysfunction and Impaired Executive Control

There is considerable evidence, which we have long acknowledged, that impairments in self-control due to cortical dysfunction, whether induced by drugs or preexisting, can play an important role in addiction (for reviews, see Jentsch & Taylor 1999, Bechara 2005, Goldstein et al. 2009, Goldstein & Volkow 2011, Bechara et al. 2019, Zilverstand & Goldstein 2020). For example, in 2000 we wrote that “repeated exposure to psychostimulant drugs may result in frontocortical dysfunction and associated cognitive deficits including impairments in decision-making and judgement,” which might interact with incentive sensitization, and “thus, in the addict, drugs may become increasingly “wanted” while at the same time the ability to make reasoned judgements about the future consequences of continued drug use becomes increasingly impaired” (Robinson & Berridge 2000, p. S106). Nevertheless, we maintain that intense motivational “wanting” to take drugs, produced by incentive sensitization, is the primary source of addictive urges that require restraint by frontocortical executive control systems.

## 5. CRITIQUES OF INCENTIVE-SENSITIZATION THEORY

### 5.1. Do Self-Administered Drugs Induce Incentive Sensitization?

The earliest studies of drug-induced sensitization used drugs that were administered to animals by an experimenter. It is important, therefore, that subsequent animal studies soon confirmed that drug self-administration also produced psychomotor, incentive, and dopamine sensitization (Robinson & Berridge 2000). However, some investigators argued that addiction-like behavior can only be produced in animals after dependence has been induced by self-administration of very large amounts of a drug like cocaine, continuously consumed for at least 6+ hours a day (sometimes called Long Access, or LgA, self-administration) (Ahmed & Koob 1998). Earlier self-administration studies showing sensitization only allowed rats to consume cocaine for 1–3 hours a day (Short Access, or ShA). Thus, in critiquing IST, Vanderschuren & Pierce (2010, pp. 183–84) wrote regarding LgA studies that “two hallmark features of behavioral sensitization are absent following excessive drug self-administration…[that is,] augmented psychomotor stimulant effects of drugs and an increased dopamine overflow in nucleus accumbens.” If that assertion were true, it would be a serious problem for IST. However, subsequent evidence has shown that LgA self-administration does cause both dopamine sensitization (Samaha et al. 2021, Alonso et al. 2022) and psychomotor sensitization (Ferrario et al. 2005, Carr et al. 2020) when rats are tested 30 days after the drugs are stopped. In short, tolerance and withdrawal effects may briefly mask sensitization effects after consuming very large quantities of drug during the first few days or weeks after LgA is suddenly terminated, but those soon subside when previously induced sensitization is revealed and then remains long-lasting (Dalia et al. 1998). This may contribute to the so-called incubation of craving phenomenon, in which drug craving rises after 1 month of abstinence to a higher level than in the first few days of withdrawal, even as tolerance and withdrawal fade (Grimm et al. 2001, Shalev et al. 2002).

Furthermore, most human drug users do not consume drugs continuously for over 6 hours per day, day after day, but rather tend to take repeated, shorter intense binges. Recent animal studies have aimed to model this binge-pause-binge pattern of human consumption more realistically. These use what are called Intermittent Access (IntA) self-administration schedules, where rats are allowed to quickly take cocaine doses as high as they choose, before a pause is interposed until the next binge. This binge-like intermittent pattern of cocaine consumption produces repeated spikes in brain cocaine concentrations and also produces marked psychomotor, incentive, and dopamine sensitization in rats as well as more addiction-like behavior than either ShA or LgA procedures (for reviews, see Allain et al. 2015, Kawa et al. 2019, Samaha et al. 2021). Similar findings have been reported after IntA self-administration of opioids and of alcohol (Samaha et al. 2021). In summary, mesolimbic sensitization is produced by a wide range of drug exposure procedures in animal studies, and the procedures that best mimic patterns of use by humans produce the strongest sensitization of mesolimbic dopamine systems and the strongest motivation to obtain drugs in animals.

### 5.2. Do Drugs of Abuse Induce Mesolimbic Sensitization in Humans and Other Primates?

Most neuroscience studies of mesolimbic sensitization have used rodents, and, as noted by Leyton (2022, p. E148), “in some circles, it remains controversial whether [sensitization effects] occur in primates.” Fortunately, there is now a growing literature, as well as experimental studies in both humans and other primates, demonstrating that limbic hyperreactivity is elicited by drug cues in people with addiction, as expected by IST, and greater sensitized mesolimbic hyperreactivity may predict greater risk of relapse (MacNiven et al. 2018, Gonzalez-Marin et al. 2020, King et al. 2021, D’Amour-Horvat et al. 2022). Thus, as Leyton’s (2022, p. E149) recent review of this issue concludes, “despite occasional claims to the contrary, there is overwhelming evidence of stimulant drug–induced behavioural sensitization in both human and nonhuman primates” and “there is compelling evidence of dopamine sensitization in primates,” including humans (see also Weidenauer et al. 2020).

### 5.3. Attentional Bias: The Salience Aspect of Incentive Salience

Many studies report that reward cues become perceptually salient, in the sense that they can capture attention in humans, even involuntarily, and that attentional capture is due at least in part to mesolimbic influences on visual neural processing (Hickey et al. 2010a,b; Watson et al. 2019; Pearson et al. 2020; Kim et al. 2021; De Tommaso & Turatto 2022). In people with substance use disorders, drug cues are particularly effective in capturing visual attention (Field & Cox 2008, Leeman et al. 2014, Bollen et al. 2020, Agarwal et al. 2021). This capture appears related to limbic hyperreactivity, it can be exacerbated during stress or craving, and the strength of attentional capture by drug cues may predict individual susceptibility to relapse. For example, stronger attentional bias to heroin cues in heroin users is reported to predict greater likelihood of relapse within the next 3 months (Marissen et al. 2006). Dopamine depletion reduces attentional capture by alcohol cues, supporting a mesolimbic influence (Elton et al. 2021). For cocaine, more years of use are also associated with greater cue-evoked activation of ventral striatum and related brain regions (Prisciandaro et al. 2014) as well as a greater chance of future relapse (MacNiven et al. 2018). Similarly, food cues capture attention in obese individuals with binge eating disorders (Woo et al. 2023), and it is likely that cues for other behavioral addictions will turn out to capture attention of individuals with those addictions, too (Anselme & Robinson 2020). Relevant to converting attention into action, attentional capture by drug cues and other reward cues is often accompanied by tendencies to approach such cues and engage in related actions (see Wiers et al. 2021).

Reward cues attributed with incentive salience can also take on attractive “motivational magnet” properties in animals, eliciting an approach that brings an animal into close proximity to the cues, called sign-tracking (Robinson & Flagel 2009, Anselme & Robinson 2020). Similar motivational magnet phenomena in humans can lead children to approach and touch reward cues or drug users to “chase ghosts”—for example, seeking out pebbles bearing resemblance to drug clumps (Rosse et al. 1993). Cue approach (sign-tracking) is dopamine-dependent and accompanied by mesolimbic neural activations (Flagel et al. 2011, Saunders & Robinson 2012, Yager et al. 2015), in contrast to goal-tracking, which refers to cue-initiated approach to the location of reward delivery (the goal) rather than the cue itself. Sign-tracking rats also show diminished prefrontal cortical top-down control over attention and executive function related to reduced prefrontal acetylcholine neurotransmission, compared to other rats (goal-trackers) that do not sign-track (Sarter & Phillips 2018), and in humans, the individual propensity to sign-track has been associated with externalizing behavior and impulsivity (Garofalo & di Pellegrino 2015, Schettino et al. 2022, Colaizzi et al. 2023). In short, incentive salience attributed to reward cues can produce attentional capture and motivational attraction to those cues (Wiers et al. 2021). Heightened incentive salience, magnified further by mesolimbic sensitization and combined with poor executive control over behavior and impulsivity, is consistent with heightened vulnerability to addiction and relapse, as viewed by the IST.

### 5.4. Does Incentive Sensitization Contribute Only Early in the Development of Addiction?

Some advocates of opponent process theory have argued that “the phenomenon of sensitization of incentive salience may be important early in the addiction process but may not be a key mechanism in later stages of addiction” (George & Koob 2017, p. 220). This is quite opposite from our view that incentive sensitization, once induced, is very persistent and contributes to relapse even after long abstinence. We believe that, when these critics make such assertions, they may have lost sight of the difference between dopamine’s roles in “wanting” versus liking. They presume that mesolimbic dopamine mediates drug positive reinforcement or liking, which we all agree is not increased by incentive sensitization. Because they view addiction as being dominated by withdrawal feelings and the desire to escape distress, in the opponent-process sense described earlier, they conclude that any role of drug liking must fade in later stages of addiction (George & Koob 2017). By contrast, we stress that mesolimbic sensitization, once induced, is highly persistent, amplifies drug “wanting” but not liking, and has been shown to last for years in both rats (Paulson et al. 1991) and humans (Boileau et al. 2006).

However, another reason these critics may believe sensitization to be short-lasting is that, in the short term, tolerance induced by heavy drug use can temporarily mask the expression of sensitization for several days to several weeks when the drugs are suddenly stopped (Paulson et al. 1991). However, sensitization reemerges at full strength within a few weeks as tolerance dissipates (Dalia et al. 1998). For example, as Dalia et al. (1998, p. 29) described, “the tolerance to cocaine was temporary while the underlying sensitization persisted,” and “the development of tolerance did not alter the underlying sensitization.” Once reemerged in this way, incentive sensitization can last years without further drug taking (Boileau et al. 2006, Samaha et al. 2021).

Similarly, in addicted humans, drug cues continue to trigger mesolimbic hyperreactivity even after decades of drug use (Koob & Volkow 2016, Zilverstand & Goldstein 2020). We believe such cue-triggered limbic hyperreactivity in addicted people is a neural signature of incentive sensitization, causing excessive wanting. Thus, to summarize the time course of neural changes in addiction more accurately, withdrawal can be powerful while it lasts but is relatively transient, fading in a few days to weeks after drug use is stopped. By contrast, incentive sensitization persists for months and years, continuing to contribute to excessive motivation for drugs, whether the sensitized person continues to take drugs or gives them up, and to cause relapse long afterward.

### 5.5. Does Drug Liking Decrease in Addiction?

A central postulate of IST is that “wanting” for drugs increasingly dissociates from liking for drugs, as sensitization and addiction develop, because only “wanting” is amplified by incentive sensitization, not liking. However, some critics have suggested that IST can be valid only if liking also simultaneously decreases (i.e., shows tolerance), as “wanting” grows via sensitization. For example, one author criticized IST on the grounds that drug liking often does not decrease in addiction, arguing that “very few human studies examining loss of pleasure have been conducted and the few that exist do not appear to support it” (Pickard 2020, p. 38). We agree that there is little evidence that drug liking usually declines in addiction, and explicitly said so in our initial paper when we wrote that “although the development of tolerance to the euphoric effects of drugs is widely accepted in the clinical literature, there is actually very little objective evidence for this” (Robinson & Berridge 1993, p. 275). We also wrote then and still believe that, “whether the subjective pleasurable effects show some tolerance or no change with repeated drug administration, the development of an addiction is still characterized by an increasing dissociation between “wanting” drugs and liking drugs” (because only “wanting” grows) (Robinson & Berridge 1993, p. 275).

However, we accept some of the blame for the misunderstanding that IST required liking to decline because figure 3 of our 1993 paper, intending to illustrate a growing dissociation between sensitized “wanting” and liking, depicted drug liking as moderately declining. That was because we wished to accommodate the views of many in the field who believed that one effect of drug tolerance was to reduce drug pleasure, largely based on observations that people often increase their doses as their addiction develops, which some presumed to be because they were getting less pleasure from the drug. However, we pointed out in 1993 that there were also alternative explanations for dose escalation even if pleasure did not decline (e.g., tolerance to the aversive or disabling effects of drugs, which would allow higher doses to be tolerated and chosen).

We reassert that IST simply posits that “wanting” grows disproportionally due to sensitization in addiction, whether liking either declines or stays the same. There is now considerable evidence that “wanting” selectively increases in addiction, and so it dissociates from liking even when liking does not decline. For example, a longitudinal study of alcohol abusers by King et al. (2021, p. 567) reported that “individuals who developed AUD over the 10-year period reported increases in wanting alcohol over time, while liking of alcohol remained high and stable.” Similar findings have been reported by others (File et al. 2022). We suspect that among people who develop addictions, some may enjoy their drugs as much as ever, including the individuals Pickard (2020) cited and the alcohol abusers studied by King et al. (2021), while some others may report a slight decline in liking, and a few may say they no longer get significant pleasure from the drugs they still want. In all these cases, however, “wanting” dissociates from liking, because incentive sensitization selectively amplifies “wanting” to the point that desire becomes dissociated from pleasure.

A related question is whether it is possible to want something that is not liked at all. Empirical evidence that “wanting” can completely dissociate from liking has been revealed most vividly in animal studies demonstrating “wanting what hurts,” powered by recruiting mesolimbic hyperreactivity to give incentive salience to a target that gives no pleasure but only pain (Warlow et al. 2020, Berridge 2023). Such “wanting what hurts” can be induced in rats by pairing optogenetic stimulation of the amygdala, which recruits mesolimbic incentive salience circuitry, with brief encounters with an electrified shock rod that delivers mildly painful shocks. Normally, rats avoid the shock rod after one or two encounters and react defensively toward it. However, amygdala-stimulated rats quickly return to hover close over the shock rod and touch it again and again despite multiple shocks, and they are willing to repeatedly climb over a barrier to reach the shock rod and to seek out shock-associated cues (Warlow et al. 2020). This creates addictive-like “wanting” even though amygdala stimulation does not enhance liking and shocks remain unpleasant (Robinson et al. 2014b, Tom et al. 2019, Warlow et al. 2020). More conventional addictive behavior results if the same amygdala stimulation is paired with pleasant targets, such as sugar or cocaine: Depending on which one is paired, it can create sugar-addicted rats that pursue only sugar and ignore cocaine or create cocaine-addicted rats that do the reverse (Warlow et al. 2020). “Wanting what hurts” provides proof-of-principle evidence for the IST postulate that intense “wanting” can become completely independent of liking for the target via neural mechanisms that produce excessive incentive salience. In a few extreme cases of human addiction, at least, “wanting” an unliked target may be possible. Mechanisms underlying “wanting what hurts” might also play a role in other clinical conditions involving the compulsive pursuit of nonhedonic targets, such as obsessive compulsive disorder or self-harming.

### 5.6. Does Incentive-Sensitization Theory Overemphasize Dopamine?

It is fair to say IST prominently features sensitization of dopamine neurotransmission. This is because even in 1993 there was evidence showing that mesolimbic dopamine projections to nucleus accumbens and related structures were key nodes in reward circuitry, and that sensitization by drugs of abuse increased dopamine neurotransmission. All that evidence has only grown. Nevertheless, we acknowledge that dopamine is only one link in the causal chain, and that other neurotransmitter systems and brain structures also contribute importantly to both drug-induced sensitization and incentive salience. For example, dopamine in nucleus accumbens and striatum interacts with glutamate signals arriving from cortex, hippocampus, and thalamus (Scofield et al. 2016, Wolf 2016). Opioid, acetylcholine, GABA, orexin, endocannabinoid, and other neurotransmitter signals also play important roles. Indeed, mesolimbic sensitization is accompanied by circuit-level changes that involve many neurotransmitter systems in many structures within mesocorticolimbic brain circuitry. Delineating how all this neural circuitry mediates sensitization and incentive salience remains an active area of research today (e.g., Pool et al. 2022, Engel et al. 2024, Fraser et al. 2023, Mohebi et al. 2023). Some of this complexity was apparent even in 1993, and so to recognize the potential for other neural elements to play a role we stated then that IST “does not require that the sole or even primary site of drug-induced neuroadaptations responsible for craving specifically be on dopamine neurons. If it is not, then our assignment of sensitization of incentive salience to dopamine would be incorrect…but it would be mediated by another, as yet unidentified neural substrate” (Robinson & Berridge 1993, p. 275, note 4). In retrospect, we believe the identification of incentive salience with dopamine was essentially correct, but of course dopamine interacts with these other neurobiological mechanisms, too.

A more fundamental challenge to our view that mesolimbic dopamine-related sensitization is a major mechanism of addiction are claims by some investigators that opioid drugs like heroin do not increase dopamine neurotransmission, or that dopamine is not necessary for opioid self-administration or addiction (Badiani et al. 2011, Nutt et al. 2015). However, there is considerable evidence that opioids indirectly increase mesolimbic dopamine release—for example, by suppressing inhibitory GABA interneurons in ventral tegmentum that normally suppress dopamine neurons, or by modulating ion channels on dopamine neurons and thus increasing dopamine neuron excitability and release. Furthermore, opioids do produce psychomotor, incentive, and dopamine sensitization (e.g., Bartoletti et al. 1983, Lett 1989, Spanagel et al. 1993). There is also evidence that mesolimbic dopamine activation is important for the motivation to self-administer opioid drugs (Johnson & North 1992, Margolis et al. 2014, Vander Weele et al. 2014, Corre et al. 2018). There may well be considerable variation in the extent to which different opioid drugs increase dopamine release, which deserves further examination, but opioid addiction appears to involve dopamine-related incentive sensitization similarly to other drug addictions (Vander Weele et al. 2014, Huang et al. 2024).

Most relevant to IST and dopamine’s role in cue-triggered wanting to take opioid drugs, cues associated with heroin and fentanyl activate mesolimbic dopamine neurons in rats (O’Neal et al. 2022) and similarly trigger fMRI hyperactivation in mesolimbic circuitry in human heroin users (Zilverstand et al. 2018, Huang et al. 2024). Furthermore, the motivational magnet properties of an opioid cue, which lead to attentional biases and sign-tracking, require dopamine and powerfully engage the mesolimbic motive circuit in animals (Yager & Robinson 2015). In sum, we conclude that cue-triggered hyperreactivity of mesolimbic dopamine-related circuitry in people with addiction is a chief driver of excessive wanting for drugs of abuse, including opioid drugs.

## 6. CURRENT ISSUES

### 6.1. Is Addiction Compulsive?

Some theorists have argued that drug use in addiction is never actually compulsive but rather is a choice like other choices (Levy 2013, Lewis 2015, Heather 2017, Pickard 2020). Whether addiction is ever compulsive hinges importantly on the precise definition of compulsion. In one traditional definition, Aristotle defined a compulsion as involving outcomes that are completely involuntary, like being pushed by an irresistibly strong wind: “Those things, then, are thought-involuntary, which take place under compulsion…in which nothing is contributed by the person…as if he were to be carried somewhere by a wind” (Aristotle, *Nicomachean Ethics* III.1). In agreement with choice theorists, few today would assert that addictive drug taking ever occurs with “nothing contributed by the person”; perhaps the closest would be Robbins’s (2019) suggestion, mentioned earlier, that addictive habits are unmotivated involuntary acts like a Tourette’s motor tic. Rather than an irresistible wind, most agree that individuals with addiction can successfully resist temptation at times, may modulate drug use temporarily in response to incentives or threats, and often choose their moments to take drugs. Thus, addiction is not compulsive in Aristotle’s classic sense.

However, we believe that a different psychological sense of compulsion also exists, namely motivational compulsion, which can more accurately apply to addiction and to disorders such as obsessive compulsive disorder (Berridge 2022). A motivational compulsion acts differently from Aristotle’s strong wind: It may be resisted at certain moments yet prevail at others. A motivational compulsion does not compel one to act contrary to choice; it distorts the mechanisms of choice and carries choice with it. Motivational compulsion can remain responsive to incentives, punishments, and contexts yet eventually compel both choice and action (see also Wiers & Verschure 2021).

To imagine the nature of a motivational compulsion that anyone might be capable of experiencing, consider the case of a person subjected to prolonged starvation. Everyone wants food after a period of fasting, but severe starvation can induce a much stronger form of motivationally compulsive wanting. Starving people may begin to obsess about food, to dream of food, and to find that available food rivets attention and becomes nearly irresistible (Keys et al. 1950). This brain-generated amplification of incentive salience can happen even without starvation. For example, similar excessive wanting for food occurs in overweight people born congenitally without the satiety hormone leptin, who overeat and constantly crave food despite being obese, and whose brains show mesolimbic hyperreactivity to food cues even after a meal (Baicy et al. 2007).

A motivational compulsion may be resisted once, twice, or even many times, but perhaps relatively few individuals are able to resist every time if faced with a long series of temptations. Sensitized people recovering from addiction may be asked to resist this unusually intense (and sometimes implicit) urge a great many times, whenever drug cues are encountered or vivid imagery of drug taking arises, but a single failure to resist may lead to relapse and possibly a binge of drug use. Finally, transient states of stress or emotional excitement can further amplify sensitized cue-triggered incentive salience, possibly to an intensity that can surprise even an experienced person recovering from addiction. Motivational compulsion, produced by excessive incentive salience, is not an external wind, but it is an intensity of temptation not faced by most of us, and one that can legitimately be called compulsive in our view.

### 6.2. Behavioral Addictions

IST was proposed specifically to explain drug addiction, not so-called behavioral addictions. Yet in the past decade, increasing neuroimaging evidence has appeared that some people with behavioral addictions show a pattern of cue-triggered mesolimbic hyperreactivity and sensitized “wanting” similar to that seen in people with drug addiction. This is manifested psychologically by an attentional bias toward the relevant cues and more intense urges or craving to approach and pursue the target of the behavioral addiction than experienced by nonaddicted individuals who recreationally pursue the same end (Boffo et al. 2018). People with behavioral addictions also show greater mesolimbic activations triggered by their addiction-related cues in neuroimaging studies relative to other reward cues or to activations triggered by the same cues in other people without the behavioral addiction. For example, food cues may trigger limbic hyperreactivity in individuals who are obese binge eaters who conceivably have an eating addiction (Gearhardt et al. 2011, Devoto et al. 2018, Stice & Yokum 2021), gambling cues trigger limbic hyperreactivity in compulsive gamblers (Linnet et al. 2012, Limbrick-Oldfield et al. 2017, Anselme & Robinson 2020), and sex cues trigger hyperreactivity in individuals treated for compulsive sex or pornography addiction (Voon et al. 2014, Kraus et al. 2016, Gola et al. 2017). We now believe that some individuals who are prone to behavioral addictions may be especially vulnerable to incentive sensitization, so that mesolimbic sensitization develops endogenously over time in their interactions with the target, even without drugs, to produce their behavioral addictions.

### 6.3. Medication-Induced Behavioral Addictions in Parkinson’s Patients

One of the most compelling demonstrations that the stimulation of dopamine systems can induce addiction-like motivation in people comes from studies of the unintended side effects of some medications for Parkinson’s disease. Unmedicated Parkinson’s patients are less likely than other people to engage in drug abuse or show behavioral addictions (Kirschner et al. 2020). However, when treated with modern dopamine direct agonist medications, which directly stimulate D2/D3 dopamine receptors, up to 45% of medicated Parkinson’s patients (Corvol et al. 2018) may experience strong new urges that can sometimes produce behavioral addictions such as compulsive gambling, sex or pornography pursuit, binge eating, compulsive shopping or Internet use, and so on (Perez-Lloret et al. 2012, Witjas et al. 2012, Callesen et al. 2013, Wu et al. 2014, Corvol et al. 2018, Drew et al. 2020). Some patients also show a dopamine dysregulation syndrome (DDS), whereby patients crave and consume much more than their prescribed amounts of L-Dopa medication, sometimes visiting multiple neurologists to collect additional prescriptions (Evans et al. 2010, Loane et al. 2015). Early reports assumed these patients only craved medication when they were off medication in a withdrawal-like state, but later studies indicate that wanting for L-Dopa can continue and may even intensify when the person is on medication, just after taking it (Evans et al. 2010, Loane et al. 2015). Furthermore, DDS patients do not necessarily like their medications more than other Parkinson’s patients—they only “want” them more.

Neuroimaging evidence suggests that medication-induced DDS and behavioral addictions in Parkinson’s disease are caused by mesolimbic sensitization in vulnerable individuals. For example, L-Dopa evokes a greater increase in dopamine release in nucleus accumbens and neostriatum of Parkinson’s patients with behavioral addictions or DDS while they view addiction-related images compared to control Parkinson’s patients who take L-Dopa but do not have behavioral addictions (O’Sullivan et al. 2011). Again, this shows that some individuals are more vulnerable to sensitization than others. O’Sullivan et al. (2011, p. 976) concluded that their results were “consistent with the hypothesis that, as a result of neural sensitization in vulnerable individuals, reward-related cues are attributed with pathological incentive salience, leading to compulsive pursuit.” Further, the amount of L-Dopa-evoked dopamine release in patients with DDS was “correlated with self-reported compulsive drug wanting but not liking, and was related to heightened psychomotor activation (punding)” (Evans et al. 2006, p. 852), consistent with IST.

### 6.4. The Role of Uncertainty

Perhaps especially relevant to gambling addiction, uncertainty about whether a reward will be gained after a reward cue can magnify the incentive salience attributed to that cue and magnify the cue-triggered activation of mesolimbic dopamine systems—even though uncertainty reduces the predictive reliability of those cues (Fiorillo et al. 2003, Robinson & Anselme 2019, Singer et al. 2020). For example, an uncertain cue that predicts a reward on 50% of occasions becomes more attractive, eliciting stronger sign-tracking in rats, than a cue that predicts a reward 100% of the time (Anselme et al. 2013, Robinson et al. 2014a). In addition, uncertainty about cues and a food reward sensitizes the subsequent psychomotor response to amphetamine (Singer et al. 2012, Zeeb et al. 2017, Zack et al. 2020). Cue-reward uncertainty increases amphetamine self-administration and food seeking by animals (Mascia et al. 2019, Robinson et al. 2023), magnifies amphetamine-induced mesolimbic sensitization (Robinson et al. 2015), and alters mesocorticolimbic dopamine and glutamate systems in ways similar to that induced by intermittent exposure to drugs (Mascia et al. 2019, 2020). In humans, problematic gambling is associated with enhanced dopamine release elicited by amphetamine (Boileau et al. 2014), possibly also reflecting mesolimbic sensitization. Thus, by rendering mesolimbic circuitry hyperresponsive, uncertainty can produce sensitization-like effects, including an increase in motivation for rewards and their cues.

## 7. CONCLUSIONS

Looking back, we are surprised at the longevity of IST, originally proposed in 1993, given the pace of developments in the field. However, in our view the weight of additional evidence accumulated over 30 years has continued to support the major tenets of IST—namely, that mesolimbic sensitization of incentive salience leads to excessive “wanting” for drugs, and an increasing dissociation between “wanting” and liking. Although dopamine participates in many behavioral and psychological functions, we also think there is now a consensus that mesolimbic dopamine’s chief role in reward is to mediate reward “wanting” but not liking. Since it was originally proposed, IST theory has expanded its scope to also include behavioral addictions, attentional capture by reward cues, medication-induced compulsions, uncertainty effects, and even the paranoia of schizophrenia (Kapur 2003), among others. Of course, the precise neural circuitry mechanisms and neurochemical changes, including in dopamine systems, that mediate incentive sensitization to selectively increase “wanting” are still an active area of investigation today (Fraser et al. 2023).

We acknowledge, as we always have, that there are many other factors that promote problematic drug use besides sensitization of incentive salience, including withdrawal or distress relief, social factors, and so on. Yet, in closing, we maintain now, as we did in 1993, that incentive sensitization carries the main explanatory weight in accounting for why particular individuals become compulsively addicted in the sense of “wanting” drugs so much. IST also helps explain why addicted individuals remain prone to relapse even after months or years of drug abstinence, even if not in withdrawal or other distress, and even if they do not expect to like their drugs very much. Thus, we conclude that accumulating evidence over the past 30 years has both bolstered support for the basic tenets of IST and expanded its reach.

## SUMMARY POINTS

1. People take drugs of abuse for many reasons (pleasure, social conformity, distress relief, etc.), and most do not become compulsively addicted, although some individuals do.
2. Understanding addiction requires explaining how vulnerable individuals develop urges to take drugs that are so intense as to be arguably compulsive.
3. In vulnerable individuals, repeated exposure to potentially addictive drugs can sensitize mesolimbic dopamine (and related) systems, which amplifies the incentive salience attributed to drug cues and drug taking, making drugs more “wanted” (but not more liked) for years after the cessation of drug use.
4. The persistence of incentive sensitization explains why people with a substance use disorder can remain vulnerable to relapse long after the discontinuation of drug use, even in the absence of withdrawal or other distress, and even if the drug is not liked much anymore.
5. The expression of sensitization can be very context dependent, being preferentially expressed in contexts in which drugs have been experienced before, which helps explain why relapse often occurs when drug-associated cues and contexts are present.
6. States of stress, withdrawal, emotional excitement, and so on, can amplify sensitized incentive salience further, causing even more intense “wanting”, which helps explain why such states increase the probability of relapse and drug taking.
7. Excessive cue-triggered “wanting” due to incentive sensitization may also contribute to the development of so-called behavioral addictions such as compulsive gambling, shopping, eating, sex, and so on, including medication-induced addictive behavior in Parkinson’s patients.
8. Although many other factors, such as habits, pleasure seeking, withdrawal avoidance, impaired executive/inhibitory control, and so on, also contribute to drug use, incentive-sensitization theory may best explain why susceptible individuals become compulsively addicted and why they remain so susceptible to relapse long after the discontinuation of drug use.

## FUTURE ISSUES

1. What neural circuitry, including corticolimbic glutamate as well as mesolimbic dopaminergic circuitry, mediates incentive salience (wanting) and incentive sensitization, and how?
2. What is the relationship between psychological phenomena studied in humans, such as an attentional bias to reward cues, and related phenomena studied in preclinical studies, such as sign-tracking?
3. How do bottom-up processes, such as incentive salience attribution, interact with top-down processes, such as executive/inhibitory control, in the development of addiction?
4. How can contextual factors, which gate the ability of drug cues to trigger incentive sensitization, be better incorporated into behavioral and neuroimaging studies of addiction in humans?
5. How do the processes of incentive motivation, affect, and learning interact, at both behavioral and neurobiological levels, to produce motivated behavior?
6. How do the affective states associated with stress, excitement, withdrawal, and so on amplify sensitized incentive salience to increase cue-triggered wanting even further?
7. What are the relative roles of implicit versus explicit psychological processes in addiction?
8. What psychological and/or brain-based treatments can decrease incentive sensitization, and therefore the degree to which drugs are “wanted”, without degrading all motivated behavior?

## Abbreviations

Withdrawal (syndrome): a cluster of unpleasant feelings experienced when heavy drug use is suddenly stopped, which persist for days to weeks
Tolerance: a decrease in a drug effect (behavioral or neurobiological) produced by repeated drug exposure, which can persist for days to weeks
Sensitization: an increase in a drug effect (behavioral or neurobiological) produced by past drug exposure and which can persist for months to years
Mesostriatal: dopamine (and other) neurons that project from the midbrain tegmentum to the dorsal neostriatum or ventral striatum (nucleus accumbens)
Mesolimbic: dopamine (and other) neurons that project from the midbrain tegmentum to the nucleus accumbens (ventral striatum) and other limbic structures (e.g., amygdala)
Liking: the subjective experience of pleasure
“Liking”: objective positive hedonic reactions to a pleasant stimulus, measured behaviorally or physiologically, which can occur either consciously or without conscious awareness
“Wanting”: the form of desire mediated by mesolimbic incentive salience, spurring approach and consumption of an attributed stimulus
Incentive salience: a motivational process that attributes incentive features to stimuli that makes them “wanted,” thought to be mediated by mesolimbic systems
Incentive sensitization: the amplification of incentive salience to compulsive intensity, which causes excessive “wanting” for drugs or other addictive targets, thought to be due to hyperreactivity of mesolimbic systems
Psychomotor activation: an increase (speeding) of psychological processing and of movements, typically caused by psychostimulant drugs like amphetamine and cocaine
Wanting: the subjective experience of desire for a declarative goal
Craving: strong subjective feelings of desire that may accompany intense incentive salience
Habit: traditionally, an automatic stimulus-response (S-R) chain, although often used today as a label for any persistent nongoal-directed action
Goal-directed action: action performed to obtain a declarative goal and sensitive to current value of the goal, often used in contrast to habits
Behavioral addiction: pathologically intense motivation focused on nondrug targets such as food, sex, gambling, etc., which may share brain mechanisms with drug addiction
Sign-tracking: a conditioned response during appetitive Pavlovian conditioning consisting of approach toward a cue that predicts pending reward delivery at another location (cf. goal-tracking)
Goal-tracking: a conditioned response during appetitive Pavlovian conditioning consisting of anticipatory approach toward the location of pending reward delivery upon presentation of a cue that predicts reward (cf. sign-tracking)
“Wanting what hurts”: a brain-induced prototype of addictive motivation manifest as intense incentive salience, or “wanting” without liking, for a target that delivers only pain
